# Accelerating protein–protein interaction screens with reduced AlphaFold-Multimer sampling

**DOI:** 10.1093/bioadv/vbae153

**Published:** 2024-10-11

**Authors:** Greta Bellinzona, Davide Sassera, Alexandre M J J Bonvin

**Affiliations:** Department of Biology and Biotechnology, University of Pavia, Pavia 27100, Italy; Department of Biology and Biotechnology, University of Pavia, Pavia 27100, Italy; IRCCS Policlinico San Matteo, Pavia 27100, Italy; Department of Chemistry, Faculty of Science, Computational Structural Biology Group, Bijvoet Centre for Biomolecular Research, Utrecht 3584 CS, The Netherlands

## Abstract

**Motivation:**

Discovering new protein–protein interactions (PPIs) across entire proteomes offers vast potential for understanding novel protein functions and elucidate system properties within or between an organism. While recent advances in computational structural biology, particularly AlphaFold-Multimer, have facilitated this task, scaling for large-scale screenings remains a challenge, requiring significant computational resources.

**Results:**

We evaluated the impact of reducing the number of models generated by AlphaFold-Multimer from five to one on the method’s ability to distinguish true PPIs from false ones. Our evaluation was conducted on a dataset containing both intra- and inter-species PPIs, which included proteins from bacterial and eukaryotic sources. We demonstrate that reducing the sampling does not compromise the accuracy of the method, offering a faster, efficient, and environmentally friendly solution for PPI predictions.

**Availability and implementation:**

The code used in this article is available at https://github.com/MIDIfactory/AlphaFastPPi. Note that the same can be achieved using the latest version of AlphaPulldown available at https://github.com/KosinskiLab/AlphaPulldown

## 1 Introduction

Mapping protein–protein interactions (PPIs) at the whole proteome level holds significant promise for uncovering new protein functions and elucidating local and global system properties within biological systems. Within an organism, PPIs are essential for vital cellular processes, such as signal transduction, metabolic pathways, and gene regulation. Interactions between different organisms, such as host-pathogen interactions, are also mediated by PPIs and are critical for understanding broader biological mechanisms.

Traditionally, studying these interactions has been laborious and often limited by the constraints of experimental techniques. The advancement of computational tools for predicting protein structures, particularly the recent introductions of Alphafold2 (Jumper *et al.* 2021) and AlphaFold-Multimer (Evans *et al.* 2022), has significantly enhanced our ability to study PPIs.

AlphaFold-Multimer leverages the power of Graphics Processing Units (GPUs) to model PPIs, making the prediction process not only accurate but also relatively fast. Since its release, several optimized pipelines have been introduced to accelerate the computational workflow even further. These improvements include the separation of Central Processing Unit (CPU) and GPU stages to optimize processing efficiency and providing faster multisequence alignment (MSA) search options (Yu *et al.* 2022). Additionally, a number of tools have been developed to help with specific AlphaFold-Multimer (Evans *et al.* 2022) applications, including AlphaPulldown (Yu *et al.* 2022), a python package which streamlines the screening of PPIs. Despite these advancements, large-scale PPI screening remains challenging due to the substantial requirements for computing time, disk space, and GPU resources. These constraints can limit the accessibility and scalability of AlphaFold-Multimer and AlphaPulldown (Yu *et al.* 2022) for large scale studies.

To help overcome this issue, we evaluated the impact of reducing AlphaFold-Multimer sampling from the default settings of five models to a single one on its ability to recognize true PPIs. To achieve this, we developed an AlphaPulldown v.1.0-based python wrapper called AlphaFastPPi. This change aims to address several critical needs: (i) it enhances the accessibility for high-throughput PPI screenings for users with limited computational capabilities; (ii) it conserves computational resources by lowering the time required by a factor 5, which is crucial to enable more sustainable large-scale analyses; and (iii) importantly, it should do so without compromising the accuracy required for confident predictions.

We tested this reduced sampling on a dataset consisting of intra- and inter-species PPIs comprising bacterial and eukaryotic proteins. This work extends the accessibility of AlphaFold-Multimer’s predictive capabilities while reducing environmental impact and resource wastage, making it a more inclusive and sustainable method.

## 2 Methods

### 2.1 Code implementation

First, MSA and structural template features are generated using the AlphaPulldown v.1.0.4 (Yu *et al.* 2022) script create_individual_features.py on CPUs.

An *ad-hoc* pipeline, AlphaFastPPi, was developed at the time because AlphaPulldown v.1.0.0 did not support single model predictions. AlphaFastPPi, which generates a single model for each PPI on GPUs when available, otherwise CPUs are used. Since it has been demonstrated that MSA pairing does not improve AlphaFold performance, only unpaired MSAs are used to increase speed (Yin *et al.* 2022). AlphaFastPPi offers two modalities, similar to AlphaPulldown v.1.0.4 (script run_multimer_jobs.py) (Yu *et al.* 2022): pull-down and all-versus-all. In pull-down mode, users provide a list of one or more proteins as “baits” and another protein as “candidates,” which are potential interaction partners. Then, AlphaFold-Multimer evaluates each bait protein against each candidate protein, generating a single model for each pair. In all-versus-all mode, the software computes all possible combinations among the proteins in the provided list, generating a single model for each pair.

**Figure 1.**
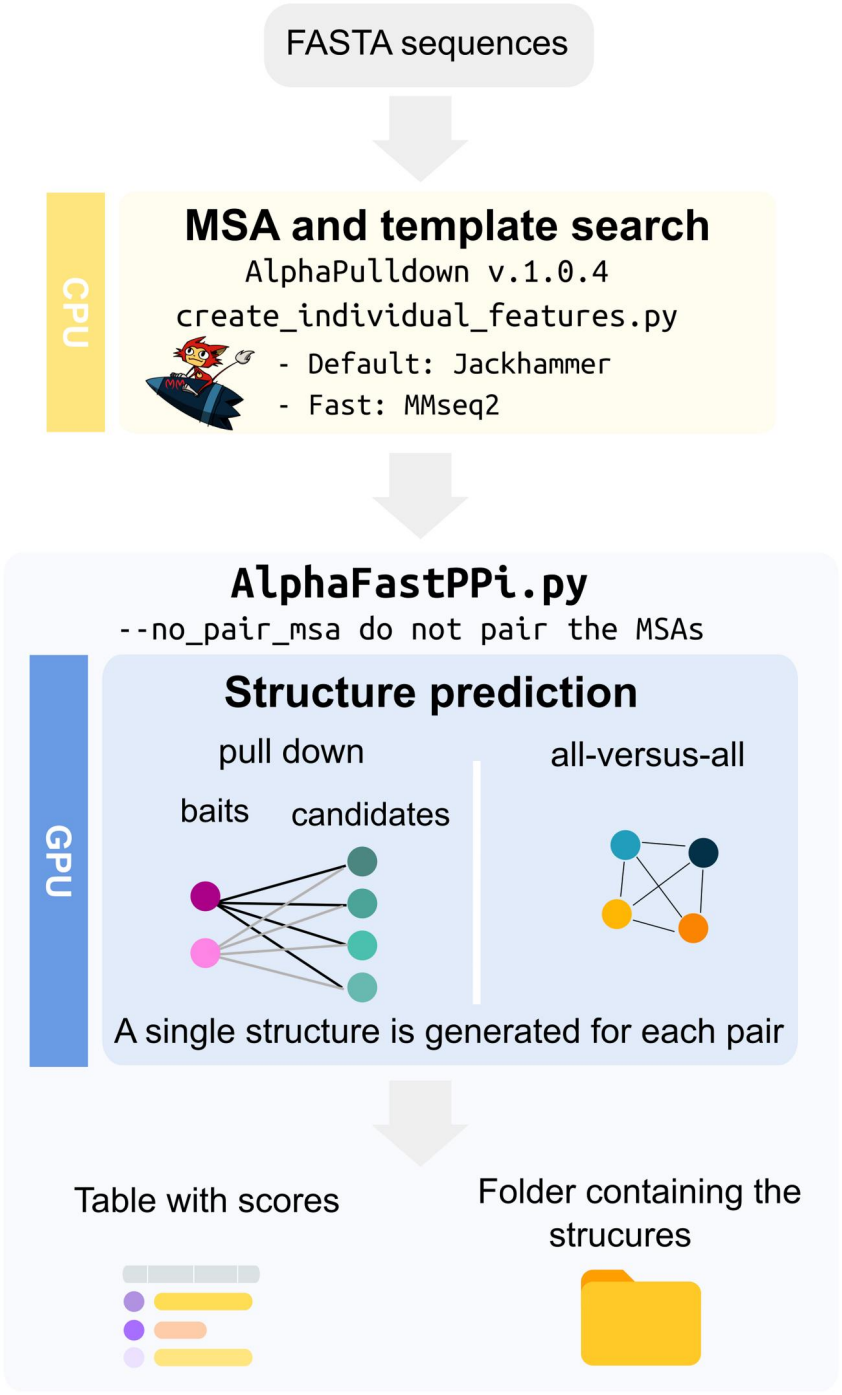
Overview of the AlphaFastPPi workflow.

After modeling, model quality scores such as pDockQ (Bryant *et al.* 2022), ipTM, ipTM+pTM, and average plDDT are calculated for each predicted PPI and the results are stored in a table for further analysis. The workflow is shown in Fig. 1.

Note that the same result can now be achieved by using run_multimer_job.py from AlphaPulldown v.1.0.4 using options—num_predictions_per_model = 1,—model_names=model_1_multimer_v3,—num_cycle = 1,—nopair_msa. For this study, we used “model_1_multimer_v3” to test the single-model system, as it allows for a low-sampling strategy and facilitates consistent comparisons. It is important to note that the five AlphaFold models, as detailed in the AlphaFold repository (alphafold/model/config.py), are not completely identical and may perform differently depending on the type of complex. While “model_1_multimer_v3” served as a representative choice to demonstrate the feasibility of a minimal sampling approach for interaction prediction, future work could explore the use of different models that may be better suited to various types of complexes, potentially offering further insights and optimization opportunities.

### 2.2 Benchmark dataset

Experimentally validated positive PPIs and a set of negative interactions taken from the Negatome database (Blohm *et al.* 2014) (Supplementary Table S1). The positive test set was composed of 286 PPIs from *Mycoplasma pneumoniae* (Elfmann *et al.* 2022) and 142 heterodimeric protein complexes between *Arabidopsis thaliana* and *Serendipita indica* (Osborne *et al.* 2023). None of the included interactions have an available 3D structure and are therefore not part of the AlphaFold training set. The negative data set consisted of an equal number (428) of true negatives composed by randomly selecting non-interacting protein pairs from the Negatome (Blohm *et al.* 2014).

## 3 Results

**Figure 2.**
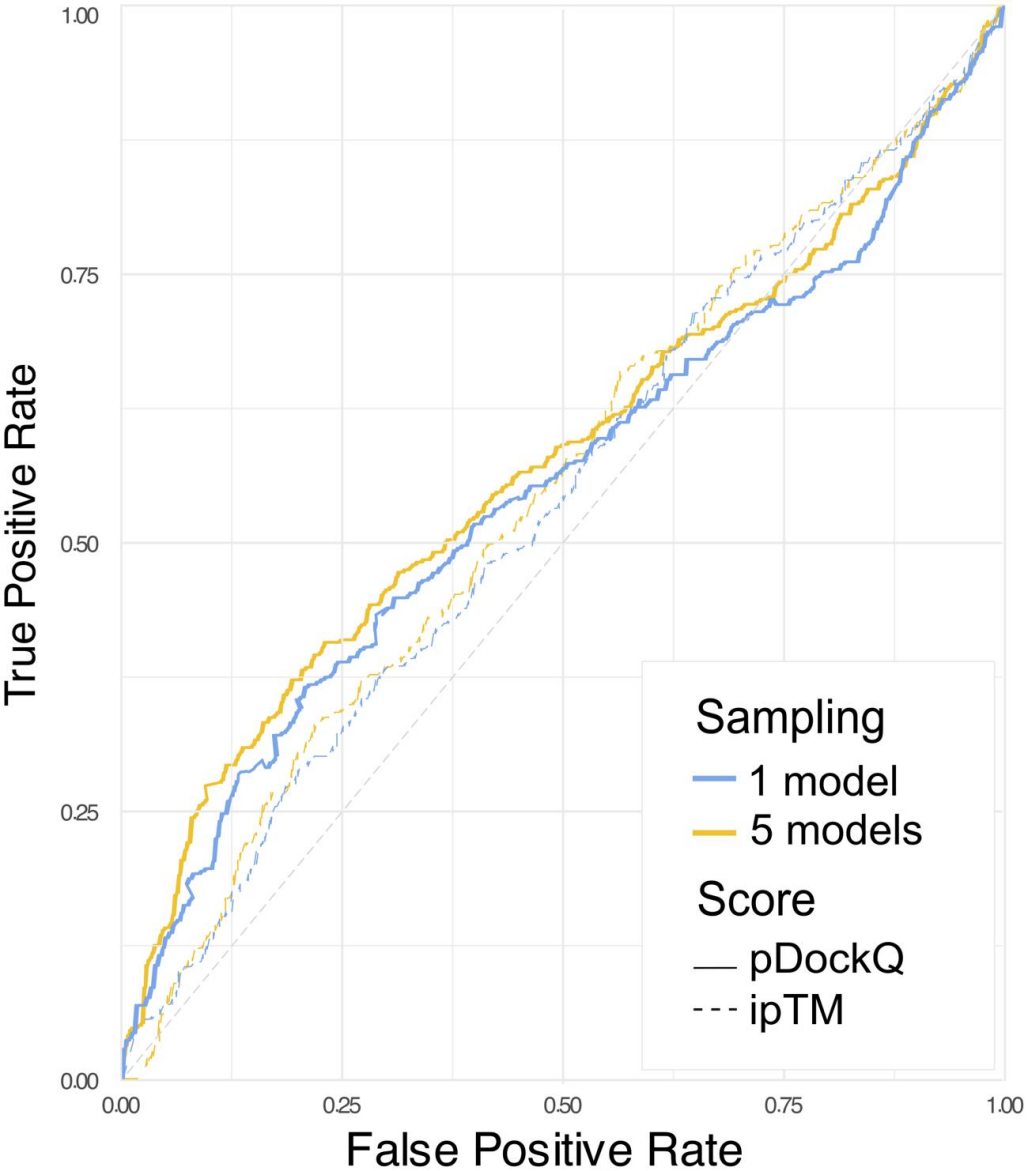
Receiver operating characteristic curve curves comparing 5 models and 1 model based on pDockQ and ipTM. The ROC curves using both the pDockQ (solid line; Area Under the Curve (AUC) “1 model” = 0.571, AUC “5 models” = 0.561) and ipTM (dashed line; AUC “1 model” = 0.539, AUC “5 models” = 0.543) scores to distinguish true from false interactions are shown. DeLong’s test was performed to compare 5 models and 1 model sampling for both ipTM and pDockQ, indicating no statistically significant difference between the AUCs (pDockQ *Z* = 0.53849, *P*-value = .428; ipTM: *Z* = 0.41, *P*-value = .328).

We evaluated the impact of reducing the sampling from the default of five models to a single model on the method’s performance in distinguishing interacting from non-interacting protein pairs. This comparison was conducted using experimentally-proven interacting and non-interacting protein pairs. All the predictions were performed on NVIDIA A100 SXM6 64GB GPUs. While GPU acceleration significantly speeds up the code, it is not a strict requirement. If no GPU is detected, the tool will automatically turn down to use CPU resources at the cost of increased computational time. As expected, reduced sampling allowed us to save approximately 4.7 times the GPU hours, translating to over 8 days in total for screening 856 interactions. This allowed also to save approximately 5.0 times the disk space (∼2 TB considering the pickled files, ∼7.5GB only considering the PDB files). Although various scoring metrics (e.g. ipTM, pDockQ), have been developed to evaluate the quality of a model, none have been explicitly designed to address this specific question of whether two proteins are interacting or not. However, among these metrics, pDockQ displayed some capability in addressing this task (Bryant *et al.* 2022). When considering default sampling (five models), only the model with the highest pDockQ among the five generated was considered to compare the distribution of values with those from collected predicting just one model for each PPI. The same approach was applied for ipTM. We did not observe any significant difference between the two methods considering both scores. This suggests that lowering the number of predictions from five to a single one does not affect the discriminatory power in distinguishing between interacting and non-interacting pairs (Fig. 2).

Consistent with previous findings (Bryant *et al.* 2022), pDockQ performed better than ipTM. When applying a pDockQ cutoff of 0.23, deemed sufficient to ensure a reasonable model quality (Burke *et al.* 2023), both AlphaPulldown and AlphaFastPPi achieved a specificity above 70%, with sensitivity around 24% on our benchmark dataset. Increasing the pDockQ threshold to 0.5 resulted in a specificity above 90%, although the sensitivity dropped below 10%. The pDockQ cutoff mentioned by Burke *et al.* (2023) should be considered as a guideline, and an appropriate threshold should be selected based on the specific dataset. More extensive benchmarking is needed to determine the optimal cutoff value. Moreover, inter-species interactions (i.e. between *A. thaliana* and *S. indica* proteins in this benchmark) showed lower confidence scores compared to intra-species interactions (i.e. among *M. pneumoniae* proteins). Additionally, we investigated whether complexes with shallow MSAs, present in both the true positive and true negative datasets, produced lower pDockQ and ipTM values. However, no direct correlation was found (Supplementary Fig. S1). It should be mentioned that massive sampling, which has been demonstrated to effectively increase the quality of models (Wallner 2023), will likely also enhance the method’s ability to distinguish between interacting and non-interacting protein pairs. However, this approach is not applicable for large-scale studies due to its substantial computational requirements. A more effective strategy could involve using a low sampling approach initially to identify potential interactions, followed by increased sampling to improve accuracy for selected cases.

## 4 Conclusions

We showed that reducing the sampling of AlphaFold-Multimer from the default value of five models to one, reduces computational resources consumption by a factor 5 while maintaining a competitive performance in predicting interacting pairs. This aligns with the needs of the scientific community, ranging from conducting quick preliminary analyses, offering a way for a rapid and effective means of narrowing down potential interactions for further investigation, to those involved in extensive studies with limited resources. Additionally, it supports extensive studies with limited resources and promotes more sustainable computational research on PPIs.

## Supplementary Material

vbae153_Supplementary_Data

## Data Availability

The code used in this article is available on GitHub https://github.com/MIDIfactory/AlphaFastPPi.
