# Supplementary material for: Accelerating protein–protein interaction screens with reduced AlphaFold-Multimer sampling
Source: Bioinform Adv. 2024 Oct 11;4(1):vbae153. doi: 10.1093/bioadv/vbae153 (PMC11513016; doi:10.1093/bioadv/vbae153)

Accelerating Protein-Protein Interaction screens with reduced AlphaFold-Multimer sampling

G. Bellinzona[1], D. Sassera[1,2], A.M.J.J Bonvin[3]

1 Department of Biology and Biotechnology, University of Pavia, Pavia, Italy
2 IRCCS Policlinico San Matteo, Pavia, Italy
3 Department of Chemistry, Faculty of Science, Computational Structural Biology Group, Bijvoet Centre for Biomolecular Research, Utrecht 3584 CS, The Netherlands

Supplementary Figure 1: Correlation between MSA depth and predicted interaction scores (pDockQ and ipTM). A) Boxplots showing the distribution of the number of sequences in the MSAs for the true positives dataset, which includes interactions between *A. thaliana* and *S. indica* (Osborne et al., 2023) and *M. pneumoniae* proteins (Elfmann et al., 2022), as well as the true negatives dataset consisting of non-interacting protein pairs from the Negatome (Blohm et al., 2014). B) Dot plots illustrating the relationship between the number of sequences in the MSA (x-axis) and the corresponding pDockQ and ipTM values (y-axis). No direct correlation was observed for either of the sampling protocols (1 model and 5 models).

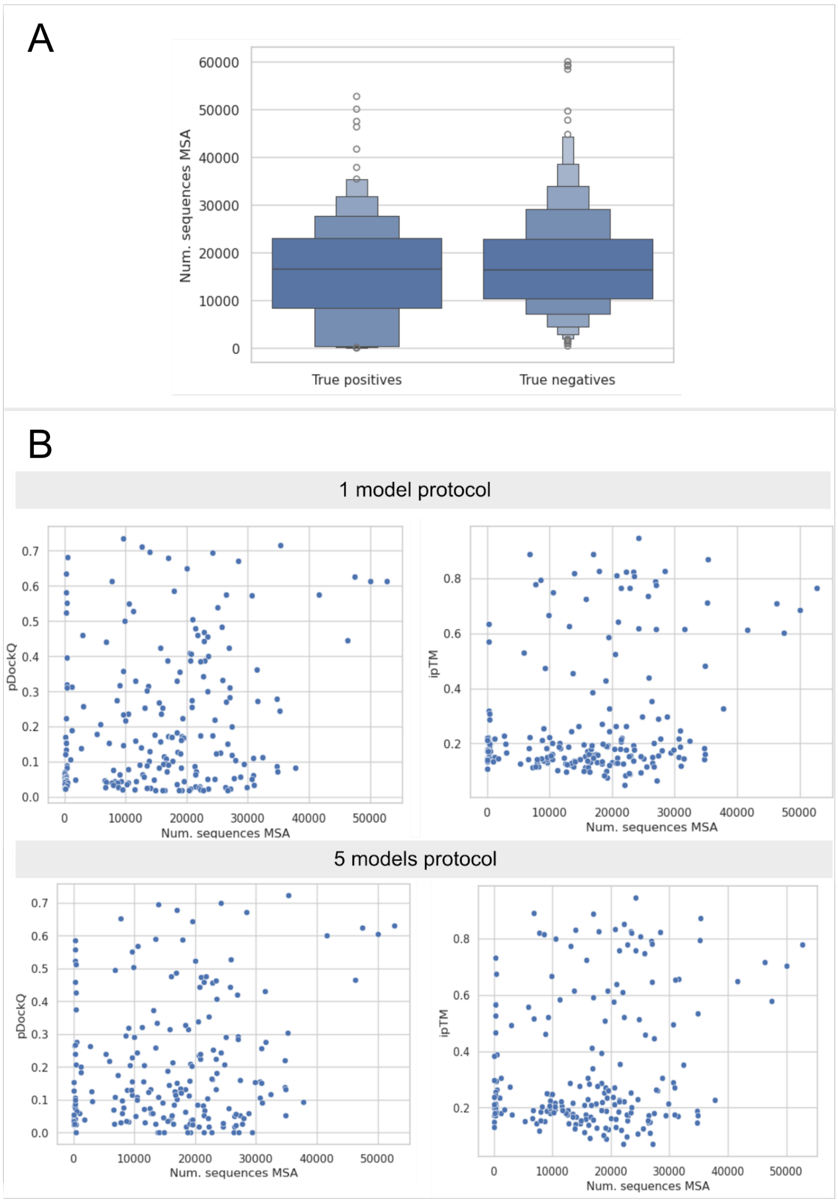

Supplement: vbae153_Supplementary_Data [file vbae153_supplementary_data.zip › Supplementary_material.pdf]
